# Cell proliferation along vascular islands during microvascular network growth

**DOI:** 10.1186/1472-6793-12-7

**Published:** 2012-06-21

**Authors:** Molly R Kelly-Goss, Erica R Winterer, Peter C Stapor, Ming Yang, Richard S Sweat, William B Stallcup, Geert W Schmid-Schönbein, Walter L Murfee

**Affiliations:** 1 Department of Biomedical Engineering, Tulane University, New Orleans, LA, 70118, USA; 2 Sanford-Burnham Medical Research Institute, La Jolla, CA, 92037, USA; 3 Department of Bioengineering, University of California-San Diego, LA Jolla, CA, 92093-0412, USA

**Keywords:** Angiogenesis, Microcirculation, Mesentery, Proliferation, Endothelial cell

## Abstract

**Background:**

Observations in our laboratory provide evidence of vascular islands, defined as disconnected endothelial cell segments, in the adult microcirculation. The objective of this study was to determine if vascular islands are involved in angiogenesis during microvascular network growth.

**Results:**

Mesenteric tissues, which allow visualization of entire microvascular networks at a single cell level, were harvested from unstimulated adult male Wistar rats and Wistar rats 3 and 10 days post angiogenesis stimulation by mast cell degranulation with compound 48/80. Tissues were immunolabeled for PECAM and BRDU. Identification of vessel lumens via injection of FITC-dextran confirmed that endothelial cell segments were disconnected from nearby patent networks. Stimulated networks displayed increases in vascular area, length density, and capillary sprouting. On day 3, the percentage of islands with at least one BRDU-positive cell increased compared to the unstimulated level and was equal to the percentage of capillary sprouts with at least one BRDU-positive cell. At day 10, the number of vascular islands per vascular area dramatically decreased compared to unstimulated and day 3 levels.

**Conclusions:**

These results show that vascular islands have the ability to proliferate and suggest that they are able to incorporate into the microcirculation during the initial stages of microvascular network growth.

## Background

Understanding the cellular dynamics involved in microvascular network growth is critical for future development of cell-specific therapies targeted at manipulating the microcirculation during tumor growth, diabetic retinopathy, myocardial infarction and other pathological conditions. Microvascular network growth in adult tissues is largely attributed to angiogenesis defined as the growth of new vessels from pre-existing vessels. Angiogenesis is commonly associated with two modes: capillary sprouting and intussusception [1]. Capillary sprouting involves the proliferation and migration of endothelial cells from an existing vessel. Intussuception is characterized by vessel splitting via the extension of endothelial cell filapodia to form an intra-lumenal pillar and subsequent lumen division. These angiogenic dynamics account for the continued remodeling and stabilization of a network [2,3].

We recently identified vascular islands, defined as endothelial cell segments disconnected from neighboring microvascular networks [4]. While vascular islands have been seen during vascular regression [5], their involvement during network growth is unknown. A potential role for blood vascular islands during angiogenesis is suggested by observations in the lymphatic system. Lymphatic vascular islands have been associated with cell proliferation, migration, and recruitment associated with lymphangiogenesis [6-9]. Based on the current evidence regarding lymphatic vascular islands during lymphangiogenesis, we hypothesize that blood vascular islands can contribute to the growth of a microvascular network during angiogenesis.

The objective of this study was to determine if vascular islands are involved in angiogenesis in the adult rat mesentery. The mesentery was selected because it allows for visualization of entire microvascular networks. We show that vascular islands are multi-cellular and associated with cell proliferation during angiogenesis. The number of vascular islands present in a network decreases during later stages of network growth. Our observations implicate vascular island proliferation and incorporation as an alternative mode of growth during the initial stages of angiogenesis in adult microvascular networks.

## Methods

### Mast cell degranulation model of angiogenesis

All experimental protocols were approved by Tulane University’s Institutional Animal Care and Use Committee. Similar to a previously established protocol [10,11], a single 2 mL dose of compound 48/80 (Sigma-Aldrich, St. Louis, MO, USA) was administered via intraperitoneal injections twice a day for 3 days in increasing concentrations (200, 400, 600, 800, and 1000 μg/mL in saline) into adult male Wistar rats (350–450 g body weight, Charles River Laboratories, Wilmington, MA, USA). Tissues were harvested and prepared for immunolabeling in three experimental groups: unstimulated (n = 4 rats), 3 days post-stimulation (n = 4 rats), and 10 days post-stimulation (n = 4 rats). The compound 48/80 inflammatory stimulus was used for this study because it produces a robust angiogenic response across the hierarchy of mesenteric networks within a relatively short time period [10-12].

### Tissue harvesting and immunohistochemistry

Mesenteric windows, defined as the thin translucent connective tissues between the mesenteric arterial/venous vessels feeding the small intestine, were labeled for BRDU + PECAM. Rats were anesthetized with intramuscular injections of ketamine (80 mg/kg bw) and xylazine (8 mg/kg bw). The peritoneal cavities were then injected with BRDU (1 mg/ml; 30 ml). After a two-hour period, the rats were euthanized via intra-cardiac injection of Beuthanasia (Schering-Plough Animal Health Corp. Union, Kenilworth, NJ, USA). 8–10 mesenteric windows were carefully dissected starting from the ileum and immediately placed in 10 mM Phosphate Buffered Saline (PBS; Sigma-Aldrich, St. Louis, MO, USA). Next, the windows were whole mounted on positively charged slides, fixed in 100% methanol for 30 minutes at −20°C, and labeled according to the following BRDU and PECAM protocol: 1) 6 N HCl for 1 hour at 37°C; 2) 1:100 monoclonal mouse anti-bromodeoxyuridine (BRDU) (Dako, Denmark) in antibody buffer solution (PBS + 0.1% saponin + 2% BSA) with 5% NGS overnight at 4°C; 3) 1:100 goat anti-mouse CY2 Fab fragments (GAM CY2 Fab, Jackson Immunochemicals, Inc., PA, USA) in antibody buffer solution for 1 hour at room temperature; 1:200 biotinylated mouse monoclonal anti-CD31 (PECAM) in antibody buffer solution at 4°C overnight; 4) 1:500 Strep-CY3 in antibody buffer solution for 1 hour at room temperature. Following each labeling step, tissues were washed every 10 minutes for 30 minutes with PBS containing 0.1% saponin. A subset of BRDU + PECAM labeled tissues was also labeled with DAPI (1:3000; Invitrogen, Molecular Probes, Eugene, OR, USA) for 10 minutes at room temperature to confirm cell nuclei.

Additional mesenteric windows from unstimulated rats were harvested, fixed with 4% paraformaldehyde for 1 hour at 4°C, and labeled for PECAM, Collagen IV (basement membrane marker), PECAM + Collagen IV, or PECAM + NG2 (pericyte marker) using colorimetric methods. PECAM colorimetric labeling has been previously described [4,13]. Collagen IV and NG2 labeling was performed using a secondary anti-rabbit primary staining kit (ImmPRESS kit, Vector Laboratories, Burlingame, CA, USA) following these steps: 1) 5-minute incubation at room temperature in 1:100 30% H_2_0_2_ in PBS; 2) 20 minute in 2.5% normal horse blocking serum (ImmPRESS kit, Vector Laboratories, Burlingame, CA, USA); 3) 1 hour incubation at room temperature with 1:1000 rabbit anti-mouse Type IV collagen Ab (Cosmo Bio Co., Tokyo, Japan) or 1:100 rabbit polyclonal Neuron-glia antigen 2 (NG2; a gift from Dr. Stallcup at the Sanford-Burnham Medical Research Institute); 4) 1 hour incubation at room temperature with horse anti-rabbit secondary agent (ImmPRESS kit, Vector Laboratories, Burlingame, CA, USA); 5) 5–10 minute incubation at room temperature with Vector Nova Red (Vector Laboratories) substrate. All labeling steps were followed by washes with PBS + 0.1% saponin, except after the Nova Red developing step, which was followed by 5-minute incubation in distilled water. For subsequent PECAM labeling, tissues were stained following a previous protocol [4,13] with the exception that the peroxidase label was developed with Vector SG (Vector Laboratories, Burlingame, CA, USA).

Positive immunostaining was confirmed by comparing labeling patterns to expected cell morphologies and to the appropriate controls: unstained, secondary antibody alone, or IgG plus secondary antibody labeled tissues.

### Image acquisition

Images were digitally captured with one of the following systems: an inverted microscope (Olympus IX70, Olympus America, Inc., Melville, NY, USA) coupled with a PixelFly camera (PCO, Kelheim, Germany) and a Olympus 4x dry, 10x dry, 20x oil or 60x oil objective; a digital camera (FUJIFILM FinePix S1 Pro) mounted on an inverted microscope (Olympus IX70) with a Cooke 5x dry, Olympus 20x dry, or Olympus 60x oil immersion objective; or on an inverted microscope (Leica DM IRE2) with a Leica SP2 AOB confocal microscopy system with a 20x dry or 63x oil objective.

### Quantification of vascular growth and cell proliferation

Two vascularized tissues were randomly selected from each rat per experimental group. Thus, a total of 8 tissues were analyzed per experimental group: unstimulated (n = 8 tissues; 2 tissues × 4 rats), 3 days post-stimulation (n = 8 tissues; 2 tissues × 4 rats), and 10 days post-stimulation (n = 8 tissues; 2 tissues × 4 rats). Network montages, generated by overlaying 4x images, were used to measure vascular area per tissue area. Vascular area was defined as the cumulative area circumscribed around every microvascular network in a tissue. Vascular length density was measured for two randomly chosen representative 4x fields of view per tissue (ImageJ, U.S. National Institutes of Health, Bethesda, MD, USA). Vascular length density was calculated by the sum of vessel segment lengths in a field of view divided by the corresponding circumscribed vascular area in that field. Vascular area and vascular length metrics were based on measurements of PECAM positive microvessels along intact networks and did not include vascular island segments.

For the unstimulated and day 3 groups, the total number of vascular islands, the number of vascular islands with at least one proliferating cell, the branch points per vascular island, the number of blind-ended capillary sprouts, and the number of capillary sprouts with proliferating cells were measured. Vascular islands were defined as blood endothelial cell segments disconnected from the neighboring blood microvascular network based on PECAM labeling. Disconnections were confirmed by focusing throughout the full tissue thickness, which is approximately 20 – 40 μm [14,15]. BRDU-positive nuclei along vascular islands and sprouts were identified based on an elongated nucleus morphology and location within the PECAM-positive vessel segments.

### Identification of vascular lumens

In additional unstimulated animals, a bolus of 2.5 mL FITC-albumin (10 mg/ml; Sigma-Aldrich, St. Louis, MO, USA) was injected via the femoral vein in adult male Wistar rats [4]. Immediately following the injection, the presence of FITC-albumin was compared to topical labeling with BSI-lectin TRITC (200 μg/mL; Sigma-Aldrich, St. Louis, MO, USA) using digital intravital microscopy. Alternatively, a 2 mL bolus of lysine fixable 40 kDa FITC-dextran (10 mg/ml; Invitrogen, Grand Island, NY, USA) was injected via the femoral vein. The presence of FITC-dextran after formaldehyde fixation was compared then to PECAM labeling along capillaries.

### Statistical analysis

Measurements are presented as means +/− SEM. Microvascular network growth metrics were compared across the 10 day time course using a one-way ANOVA or one-way ANOVA on Ranks followed by a Student-Newman-Keuls pairwise comparison test. Capillary sprout proliferation was compared between unstimulated and day 3 groups using a Student’s t-test. The vascular island proliferation and branch point metrics were compared between unstimulated and day 3 groups using a Mann–Whitney Rank Sum Test. All statistical comparisons were made using SigmaStat (Systat Software, Inc., Chicago, IL, USA). p < 0.05 was regarded as statistically significant.

## Results

**Figure 1:**
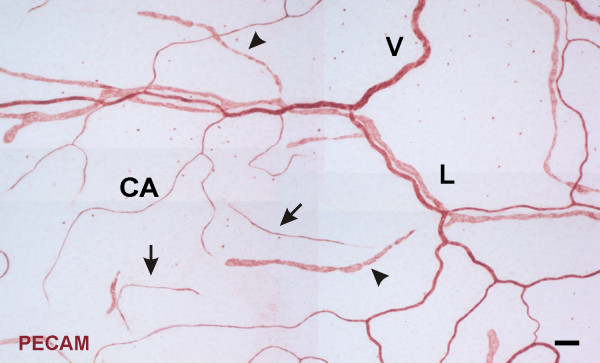
**Representative example of vascular islands in a rat mesenteric microvacular network.** Positive PECAM labeling identified lymphatic (L) and venules (V) and blood capillaries (CA). PECAM labeling also identified vascular islands defined as disconnected endothelial cell structures having either blood capillary-like (arrows) or lymphatic (arrowheads) morphology. Scale bar = 100 μm.

**Figure 2:**
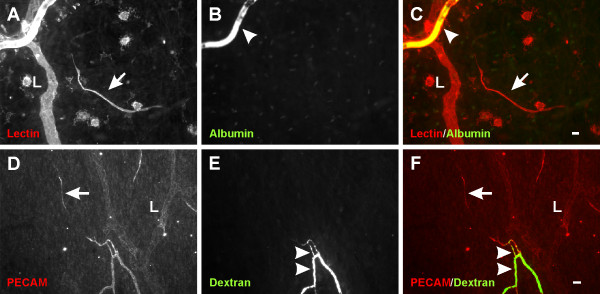
**Representative images of vascular islands without an open lumen connection to nearby vessels. A-C**) Injected FITC-albumin compared to topical lectin labeling. **D-F**) Injected FITC-dextran compared to PECAM labeling. For both cases, FITC identified patent vessels (arrowheads), but was absent along vascular islands (arrows). “L” indicates lymphatic vessels. Scale bars = 20 μm.

**Figure 3:**
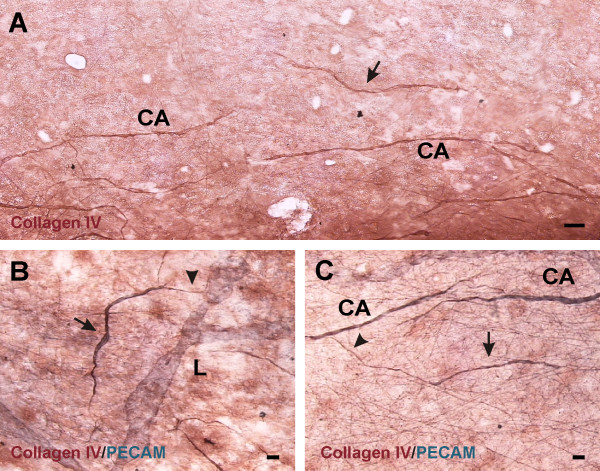
**The relation of basement membrane protein expression patterns associated with endothelial cell vascular islands (arrows) to nearby vessels.** “L” and “CA” indicate lymphatic and blood capillary vessels, respectively. Labeling with collagen IV (**A**) identifies a vascular island isolated from a nearby network of blood capillaries. Dual PECAM and Collagen IV labeling shows Collagen IV extensions (arrowheads) from isolated endothelial cells either connecting (**B**) or approaching (**C**) a nearby microvessel. Scale bars = 50 μm **(A)**, 20 μm **(B, C).**

**Figure 4:**
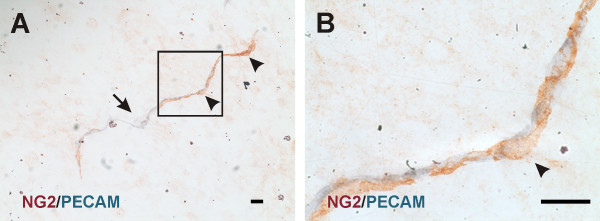
**A) Examples of NG2-positive pericytes (arrowheads) along a PECAM-positive vascular island (arrow). B)** Higher magnification view of pericyte identified by the box. NG2-positive pericytes displayed typical elongated and wrapping morphology. Scale bar = 20 μm.

In unstimulated microvascular networks, PECAM labeling served to identify endothelial cells along all hierarchies of microvascular networks down to the capillary level. PECAM-positive labeling also revealed numerous vascular islands. Vascular islands had both blood and lymphatic vessel morphologies (Figure 1). Lymphatics were distinguishable from blood microvessels by their larger diameter, their irregular vessel diameter, and a decreased PECAM label intensity along endothelial junctions [16]. Blood vascular islands displayed comparable diameters to blood capillary sprouts and lacked the lymphatic marker, LYVE-1, expression (data not shown). Vascular islands were identified as disconnected from neighboring microvascular networks based on the lack of continuous PECAM labeling as determined by focusing up and down across the full thickness of the tissue. The lack of a vascular connection to surrounding microvessels was confirmed by intravascular injection of either FITC-albumin or FITC-dextran (Figure 2). FITC was observed in all perfused microvessels, but was completely absent in nearby vascular islands. Vascular islands typically displayed no distinct collagen IV connections with nearby vessels (Figure 3), and were associated with NG2-positive vascular pericytes (Figure 4). 

**Figure 5:**
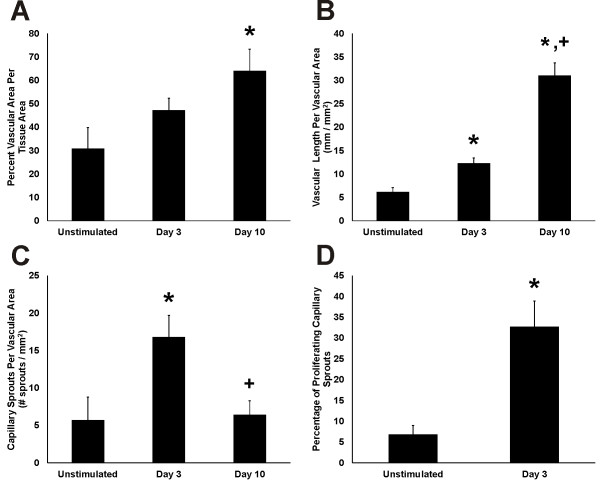
**Quantification of microvascular network growth in response to stimulation of mast cell degranulation by compound 48/80. A**) Vascular area per tissue area. **B**) Vascular length per vascular area. **C**) Total number of sprouts per vascular area. **D**) Percentage of sprouts with at least one proliferating cell. *p < 0.05 compared to Unstimulated. ^+^p < 0.05 compared to day 3.

Similar to previous reports from our laboratory [10], compound 48/80 stimulation caused mesenteric microvascular networks to undergo extensive angiogenesis (Figure 5). By day 10, networks displayed significantly increased vascular area per tissue area compared to unstimulated levels. Vascular length density increased by day 3 compared to the unstimulated level. By day 10, vascular length density further increased. The increases in vascular area and length density at day 10 were preceded by an increase in capillary sprouting. At day 3, the number of capillary sprouts per vascular area significantly increased compared to unstimulated levels. By day 10 capillary sprouting returned to an unstimulated level. During peak angiogenesis, the percentage of blind-ended capillary sprouts associated with proliferating cells was increased. 

**Figure 6:**
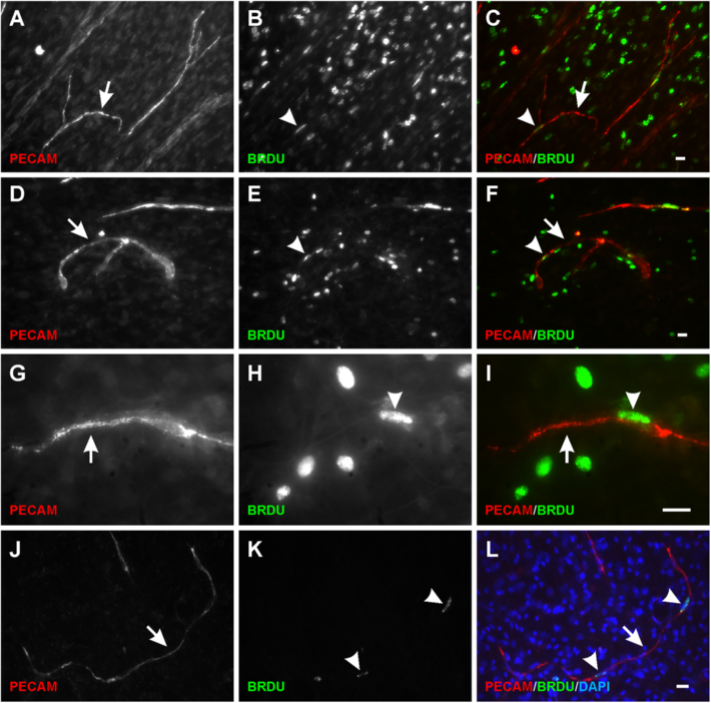
**Vascular island proliferation during microvascular network growth. A-I**) BRDU-positive cells (arrowheads) along PECAM labeled vascular islands (arrows) were observed on day 3 post compound 48/80 stimulation. Vascular islands in some cases contained multiple proliferating cells and endothelial cell branches. **J-L**) BRDU co-localized with DAPI-positive nuclei along the disconnected PECAM-positive endothelial cell segments. Scale bars = 20 μm (A-F, J-L), 50 μm (**G-I**).

**Figure 7:**
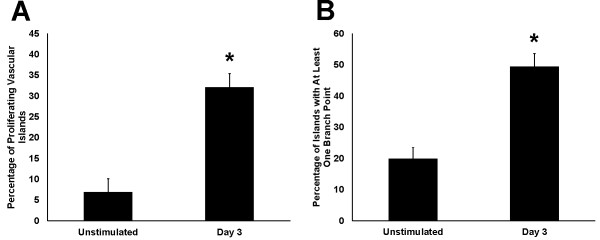
**Quantification of proliferation and endothelial cell branch points along vascular islands during microvascular network growth. A**) Percentage of vascular islands with at least one BRDU-positive cell. **B**) Percentage of vascular islands with at least one branch point. *p < 0.05 compared to Unstimulated.

**Figure 8:**
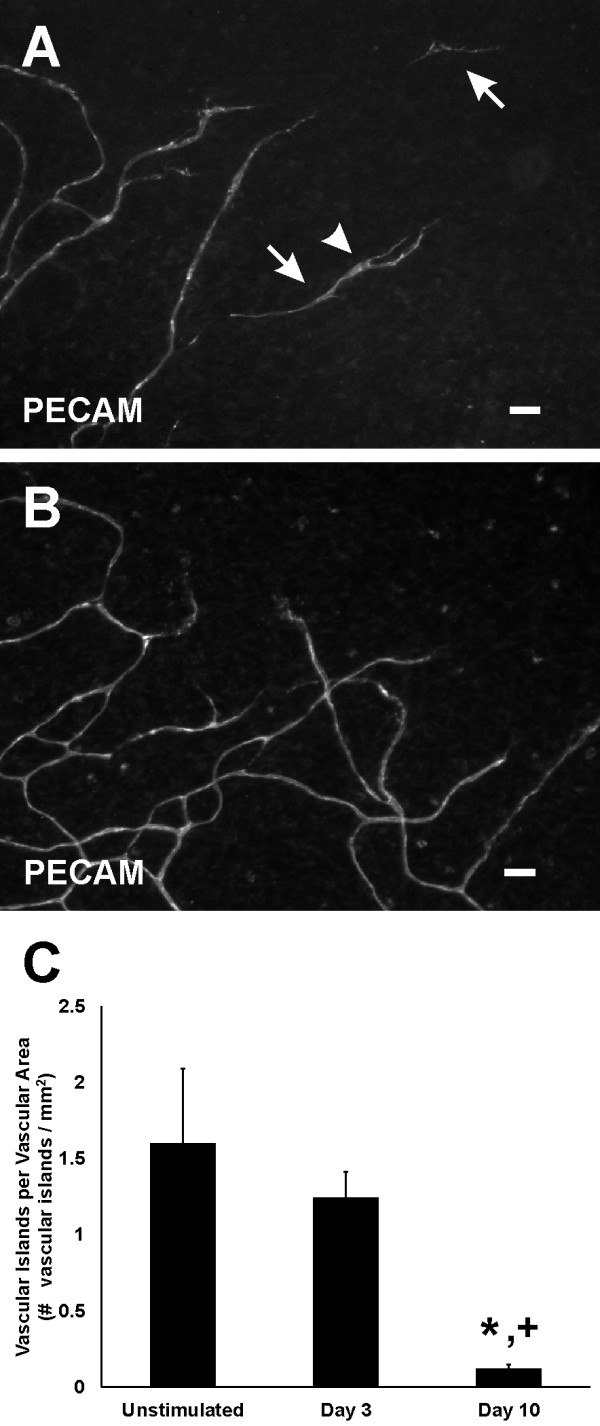
**The presence of vascular islands over the time course of microvascular network growth. A**) On day 3 vascular islands (arrows) were commonly located along the periphery of a network. Compared to the unstimulated scenario, day 3 vascular islands more frequently contained at least one branch point (arrowhead). **B**) By day 10 post stimulation, the presence of vascular islands was dramatically decreased. **C**) Number of vascular islands per vascular area. *p < 0.05 compared to Unstimulated. ^+^p < 0.05 compared to Day 3. Scale bars = 50 μm.

During the initial stages of microvascular growth, BRDU-positive cells were commonly observed along vascular islands (Figure 6). On day 3, the percentage of blood vascular islands associated with at least one BRDU-positive cell increased 5 fold compared to the percentage in unstimulated tissues (Figure 7). Blood vascular islands often contained multiple endothelial cells. As another indicator of growth, the percentage of vascular islands with branch points increased during angiogenesis (Figure 7). The location of proliferating cells within a vascular island or capillary sprout was confirmed by relative positions of BRDU-positive nuclei and PECAM labeling in 1 μm optical sections using confocal microscopy (data not shown). Proliferation was not evaluated along vascular islands at day 10 after stimulation since at this time point the number of vascular islands had dramatically decreased and too few were available for analysis (Figure 8).

## Discussion

The primary findings from this study are that 1) during the initial stages of angiogenesis vascular islands undergo proliferation comparable to capillary sprouting and 2) during later stages of angiogenesis vascular islands are no longer present, in line with the hypothesis that these segments are able to incorporate into growing microvascular networks. In addition, vascular islands are associated with vascular pericytes, which have been attributed to play a role in capillary growth and stabilization [17].

The mesentery was selected for this study because it allows for observation of intact microvascular networks (as compared to tissue cross-sections) with a resolution down to the single cell level. Use of the mesentery to investigate cellular dynamics during angiogenesis has served to identify endothelial cell phenotypic changes along capillary sprouts [18,19], the relative positioning of pericytes along capillary sprouts [20], and angiogenic pericyte phenotypes [10,11]. The current study takes advantage of a robust model of angiogenesis stimulated by injections of compound 48/80, a mast cell degranulator [12]. In the original description of this model, Norrby et al. demonstrated the ability of compound 48/80 to dramatically increase vascularized area, vascular density, and the number of vessels [12]. The quantification of angiogenesis metrics in the current study also demonstrates these dramatic effects on microvascular network growth. Our characterization of vascular islands at different time points during growth further demonstrates the usefulness of this model and suggests a potential new mode of angiogenesis in an adult tissue involving endothelial cell proliferation and incorporation.

The origin of the endothelial cells along vascular islands is currently unknown emphasizing the need for future lineage studies. Potential sources could be attributed to the migration from existing vessels, a resident population of endothelial precursor cells, or vascular regression. Support for regression is provided by the observation of increased vascular islands during vascular pruning. In rat juvenile retinas hyperoxia was shown to increase the number of disconnected vascular segments. These segments were associated with endothelial cell apoptosis [5]. In contrast, the vascular islands in rat mesenteric networks exhibited no evidence for positive TUNEL labeling (data not shown). Regardless of their origin, our results suggest that vascular islands can be triggered to enter a proliferative state.

Since proliferation along vascular islands was assessed with a single BRDU pulse, only the cells in S-phase at the time of tissue harvesting were captured. At day 3, the percentage of vascular islands with at least one proliferating cell was 33%. This value most likely underestimates the cellular proliferation associated with the vascular islands given that cells along the vascular islands are presumably in different stages of the cell cycle. Capillary sprouting is generally associated with endothelial cell proliferation. During peak capillary sprouting, the percentage of sprouts with a BRDU-positive cell was comparable to the percentage of vascular islands with a BRDU-positive cell. Thus, cells along vascular islands undergo proliferation similar to cells along capillary sprouts.

Previous dye injection experiments in the rat mesentery [21] confirmed that capillary sprouts have lumens and that endothelial cell segments extend well past them. The structure of blood vascular islands is similar to these extending endothelial cell segments. Based on this and the dye injection data presented in this study, we hypothesize that vascular islands do not form lumens prior to connection to a nearby network.

A limitation of the current study is that individual vascular islands were not tracked over the time course of angiogenesis in the same tissue. This type of time lapse investigation is required to confirm the fate of vascular islands, to determine whether vascular islands increase their length, or whether the number of endothelial cells increases along a vascular island. In our current study, the average length of a vascular island was not different between the unstimulated and 3 days post stimulation scenario (data not shown). This lack of difference can be attributed to a high variability in both vascular island length and cell number. In spite of this heterogeneity, we do report that vascular islands form new branch points during angiogenesis. The increase in cellular extensions indicates that the vascular islands are dynamic and capable of changing their structure.

The increase in cellular extensions during angiogenesis combined with the increase in cell proliferation and the drastic reduction in number results provide strong support that vascular islands are able to incorporate into growing microvascular networks. However, how these vascular islands contribute to network growth remains unclear. Future experiments will be required to determine whether vascular islands actively direct the growth of nearby vessels or whether vascular islands represent a significant cell source for new vessels in an expanding network.

The ability of vascular islands to connect to a surrounding vascular network is supported by the endothelial cell dynamics observed during embryonic vasculogenesis. Progenitor cells aggregate and elongate into chord like formations subsequently producing vascular islands composed of endothelial cells [22]. Over time these islands connect with each other and eventually to the surrounding vasculature, highlighting the ability of disconnected endothelial cell segments to connect to an existing network [22,23]. In the adult, circulating endothelial progenitor cells (EPCs) have been suggested to incorporate into remodeling vessels [24-26]. Additionally, microvessel fragments isolated from multiple adult tissues *in vitro* are able to develop intact networks after implantation [27,28]. These examples combined with our observations that the number of vascular islands decreases as vascular area and density increase support the hypothesis for incorporation of vascular islands into growing adult networks.

The involvement of vascular islands in angiogenesis is in line with a similar growth mechanism seen in lymphangiogenesis. Lymphatic vascular islands, identified as lymphatic endothelial cell segments disconnected from the surrounding network have been associated with proliferation, migration, and recruitment of cells during lymphangiogenesis [8,9,29]. These lymphatic islands have the ability to eventually connect with the existing lymphatic network in which they are located [9,29]. The overlapping mechanisms of lymphangiogenesis and angiogenesis include common cell phenotypes and responses to growth factors [9,30]. The involvement of lymphatic islands in lymphangiogenesis implicates a role for blood vascular islands in angiogenesis.

## Conclusions

Vascular islands, defined as endothelial cell segments disconnected from nearby microvascular network, are associated with cellular proliferation and increased branching during angiogenesis in the adult rat mesentery. In addition, the number of vascular islands decreases as microvascular area and length density increase. While future studies are required in other tissues and during other angiogenic scenarios, our results implicate a new mode for adult microvascular network growth involving vascular island proliferation and incorporation. The next step in confirming that vascular islands contribute to network growth requires a longitudinal study in the same tissue that allows for comparing the location of a vascular island before and after angiogenesis.

## Abbreviations

PECAM: Platelet endothelial cell adhesion molecule; PBS: Phosphate buffered saline; BRDU: Bromodeoxyuridine; BSA: Bovine serum albumin; NG2: Neuron-glia antigen 2.

## Competing interests

The authors declare that they have no competing interests.

## Authors’ contributions

MRK-G and ERW were responsible for tissue harvesting, immunohistochemistry, imaging, data collection, statistical analysis, and writing of the manuscript. PCS, MY, and RSS performed 48–80 injections and contributed to the tissue harvesting and immunohistochemistry. GWS-S contributed to the interpretation of the data and the writing of the manuscript. WBS contributed immunohistochemical reagents that led to the initial observation of the vascular islands and to the interpretation of the data. WLM conceived the study, and contributed to imaging, data analysis, and writing of the manuscript. All authors read and approved the final manuscript.
